# The Temperature Dependence of the Hexagonal Boron Nitride Oxidation Resistance, Insights from First−Principle Computations

**DOI:** 10.3390/nano13061041

**Published:** 2023-03-14

**Authors:** Liubov Yu. Antipina, Liubov A. Varlamova, Pavel B. Sorokin

**Affiliations:** Laboratory of Digital Material Science, National University of Science and Technology “MISIS”, 4 bld.1 Leninsky Av., Moscow 119049, Russia

**Keywords:** boron nitride, oxidation, BNO, DFT calculations

## Abstract

In this work, we studied the oxidation stability of h−BN by investigating different variants of its modification by −OH, −O− and −O−O− groups using an atomistic thermodynamics approach. We showed that up to temperatures of ~1700 K, oxygen is deposited on the surface of hexagonal boron nitride without dissociation, in the form of peroxide. Only at higher temperatures, oxygen tends to be incorporated into the lattice of hexagonal boron nitride, except in the presence of defects N_v_, when the embedding occurs at all temperatures. Finally, the electronic and magnetic properties of the oxidized h−BN were studied.

## 1. Introduction

Obtaining graphene in 2004 established a new field of 2D materials, which includes hundreds of atomically thin films at the moment [1]. h−BN is one of the most attractive two−dimensional structures because of its remarkable properties, including excellent optical (high transparency, strong cathodoluminescence emission, etc.) and mechanical (highest stiffness and flexibility) properties, high thermal conductivity as well as high thermal and chemical resistance [2,3,4,5,6]. BN nanoplates can retain their atomic structure up to 1150 K [7], undergoing oxidation at high temperatures with the formation of B_2_O_3_ [8]. Therefore, h−BN sheets are potentially widely used in many applications, such as long-range ultraviolet light-emitting diodes, field emitters, and polymer matrix nanocomposites operating under extreme conditions [9,10,11,12,13].

One of the important features of graphene is its easy functionalization, which gives access to its derivatives with different properties. For example, hydrogenated graphene (graphane) [14,15,16], fluorinated graphene (fluorographene) [17,18], 2D diamond films [19], as well as graphene oxide [20,21] attract attention largely. This variety of structures based on graphene distinguishes it from hexagonal boron nitride, whose derivatives are much less available. This can be explained by the polar nature of the B−N bonding [8], which favors the preferential embedding of the reference atoms rather than their adsorption. However, alternatively, introduction of oxygen atoms into a h−BN lattice provides a new practical route for its band gap and magnetic properties engineering [3]. For example, whereas in the case of graphene, oxidation occurs by the adsorption of oxygen-containing groups [20,21], hexagonal boron nitride surface treatment by oxygen usually shows only embedded oxygen. Thus, hexagonal boron nitride obtained at 1350 K and treated with atomic oxygen shows no surface oxygen but a response typical for the substitutional defect [8]. Indeed, the embedment of oxygen atoms into the h−BN honeycomb-like lattice has been confirmed under atomic resolution using annular dark-field scanning transmission electron microscopy [22]. Additionally, it was found that the optical band gap of h−BN nanosheets is reduced to 4.31 eV, and the conductivity is improved by 2 orders of magnitude after oxygen plasma treatment [23]. In Ref. [24], the narrowing of the optical bandgap in h−BN nanosheets until 2.1 eV was shown and the appearance of paramagnetism and photoluminescence (of both Stokes and anti−Stokes types) in them after oxygen doping and functionalization was observed.

It was also demonstrated that the perfect structure of hexagonal boron nitride affects its stability against oxidation. Highly oriented h−BN nanosheets have a better thermal stability compared to randomly oriented sheets [25]. This difference can be attributed to multiple defects occurring at the folds and edges of the h−BN sheets. In Refs. [26,27], similar results were obtained for the adsorption of oxygen with its embedding in the lattice on the h−BN surface at temperatures above 600 K. It has also been shown that oxygen can heal nitrogen defects by embedding itself in the lattice of hexagonal boron nitride [26,27]. In addition, several predictions using evolutionary algorithms [28,29] show that in the presence of multiple vacancies in the lattice of hexagonal boron nitride, oxygen is possible to heal these vacancies, transforming into a sufficiently distorted but more stable form B_5_N_3_O_2_ [28], which is able to halt its further oxidation as well.

Nevertheless, there is some evidence of the possibility of h−BN surface functionalization. In Refs. [30,31,32,33], it was shown that during adsorption of molecular oxygen on the hexagonal boron nitride surface on Ni(111) substrate at sample temperature 240 K, oxygen does not dissociate but binds with two boron atoms on the surface. This structure was determined as an intermediate between superoxide and peroxide. The authors also note that without additional energy from the supersonic molecular beam, oxygen adsorption was not detected [31]. In Ref. [34], it was shown that the hydroxyl group can also be successfully grafted onto the h−BN surface via low-temperature plasma treatment. In Ref. [35], the authors modified h−BN by hydroxyl groups by ordinary treatment in NaOH solution at 350 K and further drying at 380 K. All these data show that hydroxyl groups can be successfully grafted to the h−BN surface at temperatures below 400 K.

It was predicted [36,37] that the −OH group is stable on the h−BN surface, and the bonding with the boron atom is much more favorable than with the nitrogen atom. It was also determined by theoretical methods that −OH groups are preferentially adsorbed on both sides of the h−BN plane rather than on one side only. The functionalized h−BN sheet is the most stable when the degree of coverage by OH radicals reaches 60% [37]. It has also been shown theoretically that the high oxidation stability is related to the complexity of dissociation of the oxygen molecule adsorbed on the h−BN sheet [38]. Moreover, depending on the initial location of the first connected O atom, subsequent oxidation has a tendency to form an O−domain or O−chain on the h−BN sheet [38,39].

The above data allow us to make the following statements regarding the interaction of h−BN with oxygen:

1. In the presence of a nitrogen vacancy, oxygen will be embedded in the lattice at the vacancy site;
2. At high processing temperatures or when treated with atomic oxygen, it is also able to replace the nitrogen atoms in the h−BN lattice;
3. When h−BN is treated with molecular oxygen at low temperatures, the O_2_ molecule does not dissociate and binds to the surface of h−BN as peroxide or superoxide;
−OH groups can be successfully adsorbed to the surface of hexagonal boron nitride at low temperatures, in a solution of alkali or plasma treatment;
5. Oxygen binds preferably with the boron atoms on the surface of h−BN rather than with the nitrogen atoms.

The variety of data requires the creation of a general model of hexagonal boron nitride oxidation to achieve an understanding of the processes of its interaction with oxygen. Thus, in the first part of the paper, we studied the thermodynamic basis of oxygen incorporation into the hexagonal boron nitride lattice and showed its tendency to heal nitrogen vacancies with oxygen with the formation of a B_x_N_x−1_O structure. The second part of the paper is devoted to the adsorption of oxygen on the surface of h−BN in the form of various groups (both hydrogen-free and hydroxy groups). Finally, we studied the stable structures of h−BN oxide and showed that they have a variety of electronic and magnetic properties.

## 2. Computational Details

All calculations of atomic and electronic structure of oxidized hexagonal boron nitride were performed using a density functional theory (DFT) [40,41] within the PBE−PAW approximation [42], with the periodic boundary conditions using a Vienna Ab−initio Simulation Package (VASP) [43,44]. The plane−wave energy cutoff was equal to 400 eV. To calculate the equilibrium atomic structures, the Brillouin zone was sampled according to the Monkhorst−Pack [45] scheme with a grid 4 × 4 × 1 k−points. To avoid the interaction between the neighboring h−BN layers, the vacuum space between them was greater than 15 Å. The structural relaxation was performed until the change of the total energy is smaller than 10^−6^ eV. Due to the fact that the oxygen molecule can change the electron conjugated system of h−BN, we made the spin polarized calculations. Although the DFT method is widely used to calculate electronic structure, it poorly describes the strength of dispersion and the van der Waals interactions. The Grimme correction method (DFT−D2 method) [46] was used to take into account the possible interaction with detached molecular oxygen from the surface of hexagonal boron nitride.

We designed hexagonal boron nitride structures containing 72 atoms in the hexagonal supercell (B_36_N_36_). The calculated lattice parameter for unit cell of h−BN was a = 2.51 Å, which corresponds very well with the experimental data (2.51 Å [47]). After oxygen adsorption, lattice optimization was also performed to account for cell changes during sp^3^−bond formation. Lattice changes after adsorption of oxygen-containing groups were less than 1%. This cell corresponds to the oxygen concentration of *c*~1.4 at.% (*c*, at.% = *n_O_/∑n_i_*, where n is the number of *i*−type atoms in the system), which is significantly lower than the oxygen concentration usually observed in the experiment for the production of boron nitride (4–5 at.%) [3,13,48]. This way, we are modeling the beginning of the oxidation process of the boron nitride sheet.

We investigated different types of oxygen binding to the surface of hexagonal boron nitride:

1. The direct embedding of oxygen in the h−BN lattice by replacing the nitrogen site;
2. The oxygen healing of the h−BN lattice contained nitrogen vacancy (formation of B_x_N_x−1_O composition);
3. The adsorption of oxygen onto the h−BN surface in epoxy or peroxide forms;
4. The adsorption of oxygen onto the h−BN surface as hydroxy group.

### Thermodynamic Analysis

The temperature-dependent possibility of h−B_x_N_y_O_z_ film formation is analyzed by the approach used in Refs. [49,50,51] where free energy (*γ*) is used as a main stability value. The energy (given per oxygen atom) can be defined as

(1) $$\gamma \left(T,P\right)=G_{tot}\left(T,P\right)-n_{BN}\mu_{BN}\left(T,P\right)-n_{ad}\mu_{ad}\left(T,p_{ad}^{0}\right)$$

where *G_tot_* is a Gibbs free energy per unit cell of the system; *n_BN_* and *n_ad_* are the number of BN pair and adsorbed atoms or molecular groups in the unit cell, respectively; $p_{ad}^{0}$ is the partial pressure of adsorbed atoms or molecular groups and *P* is the total pressure of the system; and *μ_BN_* and *μ_ad_* represent the chemical potentials for boron nitride and adsorbed atoms.

It was assumed that the bulk system is in equilibrium with the structure in its natural state,

(2) $$G_{bulk}\left(T,P\right)=\mu_{BN}\left(T,P\right)$$

where *G_bulk_* is the Gibbs free energy of a hexagonal boron nitride sheet per unit structure. Thus, we can rewrite the free energy as

(3) $$\gamma \left(T,P\right)=G_{tot}\left(T,P\right)-n_{BN}G_{bulk}\left(T,P\right)-n_{ad}\mu_{ad}\left(T,p_{ad}^{0}\right)$$

This equation gives γ in terms of classical thermodynamics, where the energy of formation of a molecule under standard conditions is zero. The Gibbs free energies of the slab and the bulk crystal are calculated using DFT, ignoring the temperature and pressure dependence because it is negligibly small compared to the vapor. Since this paper considers the qualitative behavior of stability rather than quantifying the absolute energy of formation, these small corrections have not been calculated [52]. The energy shows not the stability of one structure over another, but the thermodynamic yield of the reaction (exothermic or endothermic reaction) and, therefore, the probability of the selected reaction proceeding spontaneously without regard to kinetic parameters.

The γ value was estimated by DFT via the following equation, where *G_tot_* and *G_bulk_* represent the DFT total energies:

(4) $$\gamma (T)=E_{B_{x}N_{x-y}O_{y}}-E_{B_{x}N_{x}}-n_{ad}\mu_{ad}\left(T,p_{ad}^{0}\right)$$

To assign temperature and pressure values to γ values, the DFT energy should be related to the chemical potential *μ_ad_*. In the ideal gas approximation, the *μ_ad_* can be written as

(5) $$\mu_{ad}\left(T,p_{ad}^{0}\right)=\mu_{ad}\left(T,P\right)+\frac{1}{2}kTln\left(\frac{p_{ad}^{0}}{P}\right)$$

It can be reformulated by introducing a term for the change in the chemical potential when moving from *T* = 0 to *T* = *T* at constant pressure *P*:

(6) $$\mu_{ad}\left(p_{ad}^{0}\right){|}_{T=0}^{T=T}=\mu_{ad}\left(T,P\right)-\mu_{ad}\left(0,P\right)$$

Combining Equations (5) and (6), it can be obtained

(7) $$\mu_{ad}\left(T,p_{ad}^{0}\right)=\mu_{ad}\left(0,P\right)+\Delta \mu_{ad}\left(P\right){|}_{T=0}^{T=T}+\frac{1}{2}kTln\left(\frac{p_{ad}^{0}}{P}\right)$$

The value of $\Delta \mu_{ad}\left(P\right){|}_{T=0}^{T=T}$ can be deduced from thermodynamic tables [53]. Values in thermodynamic tables a corrected to 0 K as follows:

(8) $$\Delta \mu_{ad}\left(P\right){|}_{T=0}^{T=T}=H^{0}-H_{T=0}^{0}-TS^{0}$$

In analogy to Equation (5), chemical potential is defined in following way:

(9) $${\mu^{\prime }}_{ad}\left(T,p_{ad}^{0}\right)=\Delta \mu_{ad}{|}_{T=0}^{T=T}+\frac{1}{2}kTln\left(\frac{p_{ad}^{0}}{P}\right)$$

If the reaction involves not only sorption but also the release of a gas, a term $\mu_{des}\left(T,p_{des}^{0}\right)$ is added to the equations to take this desorption reaction into account. Equations (5)−(9) are similar for $\mu_{des}$ for substituted nitrogen or other gas molecules removed during the corresponding reaction.

For example, the process of replacement of nitrogen by oxygen atoms in hexagonal boron nitride can be represented as follows:

$$\begin{array}{l} 1.\;B_{x}{N_{x}}_{\left(s\right)}+\frac{y}{2}{O_{2}}_{\left(g\right)}=B_{x}N_{x-y}O_{y}+\frac{y}{2}{N_{2}}_{\left(g\right)},\\ 2.\;B_{x}{N_{x}}_{\left(s\right)}+yO_{at}=B_{x}N_{x-y}O_{y}+\frac{y}{2}N_{2},\\ 3.\;B_{x}{N_{x}}_{\left(s\right)}+\frac{3y}{2}{O_{2}}_{\left(g\right)}=B_{x}N_{x-y}O_{y}+yN{O_{2}}_{\left(g\right)}. \end{array}$$

with the following reaction energy *E_R_*:

(10) $$1.\;E_{R}^{\left(T=0\right)}=\frac{1}{y}\left(E_{B_{x}N_{x-y}O_{y}}+\frac{y}{2}E_{N_{2}}-E_{B_{x}N_{x}}-\frac{y}{2}E_{O_{2}}\right),\;2.\;E_{R}^{\left(T=0\right)}=\frac{1}{y}(E_{B_{x}N_{x-y}O_{y}}+\frac{y}{2}E_{N_{2}}-E_{B_{x}N_{x}}-yE_{O}),\;3.\;E_{R}^{\left(T=0\right)}=\frac{1}{y}(E_{B_{x}N_{x-y}O_{y}}+yE_{NO_{2}}-E_{B_{x}N_{x}}-\frac{3y}{2}E_{O_{2}}).$$

Further consideration involves the energies of simple substances and their chemical potentials according to the target reaction. It should be noted that using different reaction equations would also change *E_R_*. In this work, we considered the processes of structure formation only using simple gaseous compounds (O_2_, N_2_, H_2_), without considering the influence of other gases (atomic forms, nitrogen oxides, etc.).

Thus, we obtain Equation (4) combined with ${\mu^{\prime }}_{ad}$ from Equation (9) in the form of

(11) $$\gamma \left(T\right)=E_{R}^{\left(T=0\right)}-\frac{n_{ad}}{y}{\mu^{\prime }}_{ad}\left(T,p_{ad}^{0}\right)+\frac{n_{des}}{y}{\mu^{\prime }}_{des}\left(T,p_{des}^{0}\right)$$

This expression allows to translate the chemical potential to temperature and pressure conditions and vice versa. Note that at absolute zero temperature, ${\mu^{\prime }}_{ad}=0$ and γ = *E_R_*. The partial pressure of gases was taken to be 1 since we considered single gas molecules in the calculation.

## 3. Results

### 3.1. The Embedded Oxygen

**Figure 1:**
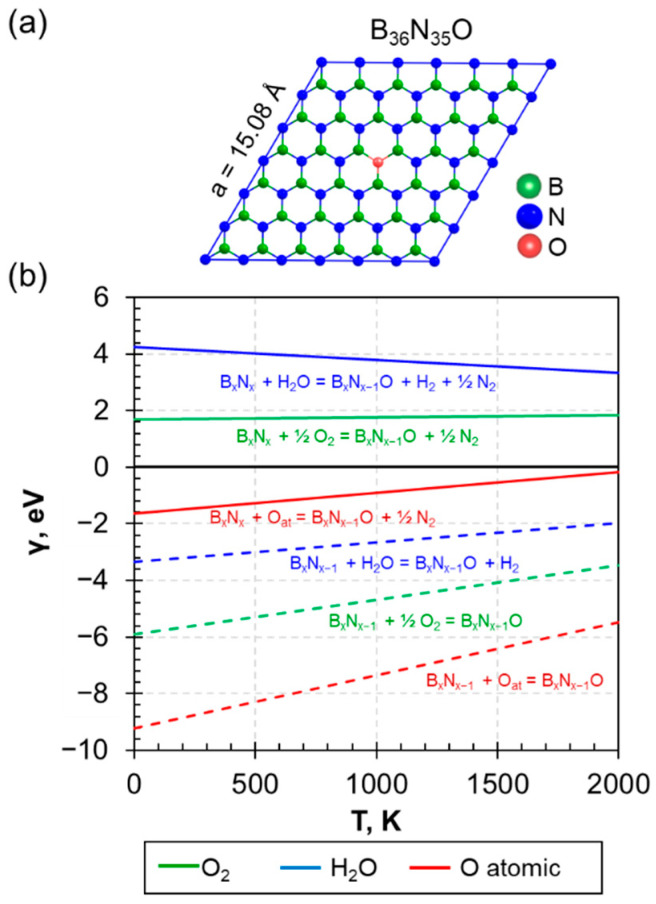
(**a**) Atomic structure of h−BN with substitutional impurity due to O atom incorporation (O_N_). Boron, nitrogen, and oxygen atoms are marked by green, blue, and red circles, respectively. (**b**) Dependence of free energies from temperature of hexagonal boron–nitrogen interaction with different sources of oxygen (blue, green, and red lines correspond to water, molecular oxygen, and atomic oxygen, respectively). The solid line corresponds to the initially perfect h−B_36_N_36_ structure; the dashed line relates to the defective h−B_36_N_35_ structure.

For the case of perfect h−BN, we proposed that the process of oxygen embedding (Figure 1a) can be described by the reactions:

(12) $$\begin{array}{c} B_{x}N_{x}+\frac{y}{2}O_{2}\\ B_{x}N_{x}+yO_{at} \end{array}=B_{x}N_{x-y}O_{y}+\frac{y}{2}N_{2};$$

(13) $${B_{x}N_{x}+{yH}_{2}O=B}_{x}N_{x-y}O_{y}+\frac{y}{2}N_{2}+{yH}_{2}.$$

The behavior of *γ* as a function of temperature is linear and presented in Figure 1b, with solid lines for perfect h−BN. It is found that the *γ* of molecular oxygen or water molecule displays positive values (Figure 1b, green and blue solid line for O_2_ and H_2_O, respectively) for the embedding of oxygen in the B_36_N_36_ lattice (Figure 1). In contrast, atomic oxygen can be relatively easily included with negative formation energy in the entire temperature range considered (Figure 1b, red solid line) with the emission of nitrogen. This result shows that the ideal structure of hexagonal boron nitride is resistant to oxidation under normal atmosphere. Oxidation by molecular oxygen-containing groups is thermodynamically unfavorable and requires additional energy costs. However, in an atmosphere of active atomic oxygen, oxidation can occur spontaneously and at low temperatures.

However, according to the reference experimental data [26,27], oxygen tends to heal the nitrogen vacancies in the BN structure with the formation of B*_x_*N_*x*−*y*_O*_y_* composition. Our computations confirm the thermodynamic favorability of such a process for all studied oxygen in the entire temperature range considered by calculating the *γ* including defective h−B*_x_*N*_x−y_* as the initial structure (see Figure 1b, dashed lines). In the presence of a N_v_ defect in the initial structure, the incorporation of oxygen is a favorable process for all considered oxygen sources; therefore, the healing of nitrogen vacancies should occur spontaneously.

This resistance to oxidation allows us to consider h−BN as a superior anti-corrosion material. Indeed, it has been shown that h−BN can protect the copper layer from oxidation much longer than graphene. Both 2D materials can increase the initial oxidation temperature of copper by more than 120 K [54]. In the short time period at temperatures below 250 K, both graphene and h−BN coatings provide excellent protection for Cu oxidation, but graphene stops working as a protective layer after only a few hours, in contrast to boron nitride [55]. Additionally, Ref. [56] compared the anti-corrosive properties of coatings made with h−BN, MoS_2_ and α−ZrP, which can isolate the corrosive medium from the substrate, providing efficient protection of metals [56]. In addition, the insulating nature and thermal barrier properties made h−BN more attractive than graphene in protective coatings [57]. h−BN proved to be the best choice for potentially solving the problem of graphene-induced microgalvanic corrosion in the long term.

### 3.2. Adsorption of Oxygen on h−BN Surface

**Figure 2:**
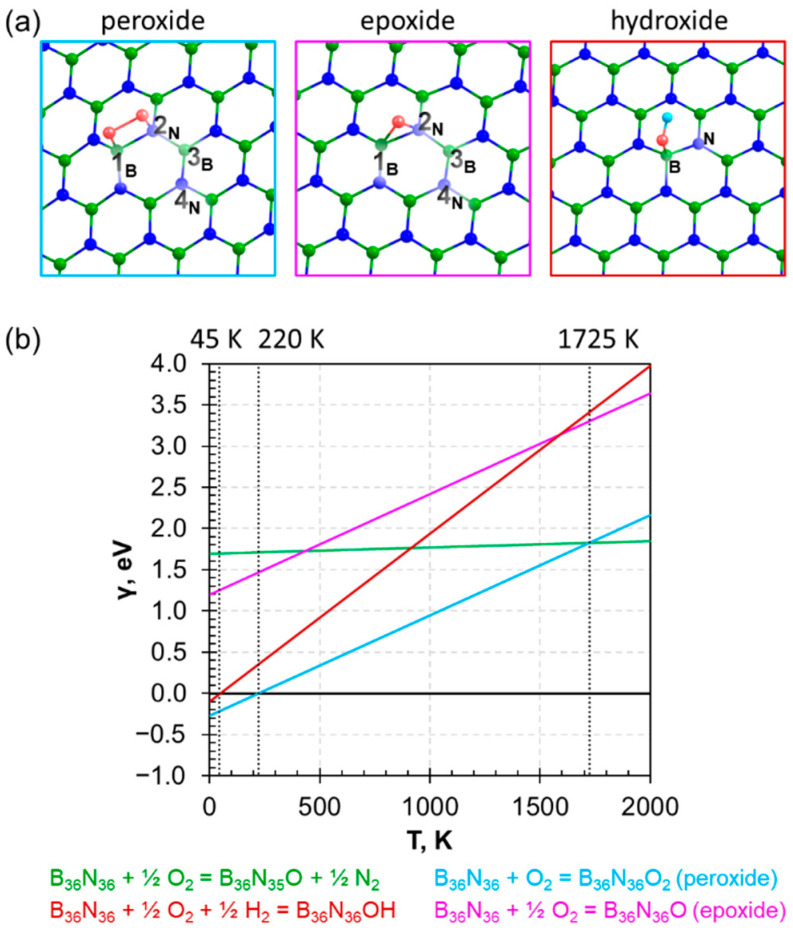
(**a**) Fragment of atomic structure of h−BN with peroxy− (**left**), epoxy− (**middle**) and hydroxy− (**right**) group in lattice. Boron, nitrogen, and oxygen atoms are marked by green, blue, and red circles, respectively. (**b**) Dependence of surface free energies on temperature. The color of the lines corresponds to the reactions in the figure below. The crossing lines and corresponding temperature indicated by vertical dotted lines.

In Refs. [30,31,32], it was shown that, due to adsorption onto the h−BN surface arranged on a Ni(111) substrate, oxygen does not dissociate, but binds with two boron atoms in the peroxide form. We considered this system by allocating oxygen in positions 1_B_−2_N_, 1_B_−3_B_, and 2_N_−4_N_ (Figure 2a). Position 1_B_−4_N_ was not considered because the distance between boron and nitrogen is too large to preserve the O−O bond. Due to optimization, the 1_B_−3_B_ and 2_N_−4_N_ structures transform into the more favorable 1_B_−2_N_ configuration discussed below. It should be noted that the stable 1_B_−3_B_ peroxide structure observed in Ref. [32] is related to the specific arrangement of h−BN on a Ni(111) substrate. When O_2_ deposits on the h−BN surface, the boron atoms interact with oxygen, while the nitrogen atoms with disrupted *sp*^2^−hybridization bind with the substrate. In the case of the freestanding h−BN, such stabilization does not occur, and the 1_B_−3_B_ configuration becomes unstable.

We considered the addition of oxygen on the hexagonal boron nitride surface by reaction

(14) $$B_{x}N_{x}+O_{2}=B_{x}N_{x}O_{2},$$

(15) $$B_{x}N_{x}+1/2O_{2}=B_{x}N_{x}O.$$

Figure 2 shows that the formation of the peroxide group can proceed spontaneously at low temperatures (up to 220 K), while sorption at higher temperatures requires additional energy input due to the endothermicity of the reaction.

The case of dissociation of the O_2_ molecule and the subsequent formation of an epoxy group was also studied. Unlike in the peroxide case, the epoxide group formation is unfavorable in the entire range of temperatures (Figure 2), which is consistent with the experimental data [30,31,32]. This confirms the fact that oxygen can adsorb onto the surface of hexagonal boron nitride without dissociation at sufficiently low temperatures. This interaction does not lead to a noticeable destruction of the lattice. The formation of the dissociated form is thermodynamically disadvantageous, as well as its further incorporation.

Next, we considered the deposition of oxygen on the hexagonal boron nitride surface in the form of a hydroxyl group −OH as described by the following reaction:

(16) $$B_{x}N_{x}+\frac{y}{2}O_{2}+\frac{y}{2}H_{2}=B_{x}N_{x}{\left(OH\right)}_{y}$$

We obtained that the OH group tends to bind to the boron atom, which is consistent with Refs. [36,37]; however, the exothermicity reaction range is quite small and requires cryogenic temperatures (T = 45 K). The adsorption of −OH on the nitrogen atom is thermodynamically unstable by reaction (16). It was found that in most cases the structure was broken during geometry optimization and transformed to more stable configurations (the −OH group either moved to the boron atom or reaches a local minimum in the intermediate state, forming an epoxy-like bond without hydrogen detachment).

Summarizing all the results presented in Figure 2, one can conclude that the adsorption of oxygen onto the surface of defect-free hexagonal boron nitride does not occur spontaneously. At sufficiently low temperatures, there is a window of exothermic adsorption of oxygen on the surface. In this case, at temperatures higher 1500 K, there will be predominantly embedding of oxygen in the lattice with replacement of the nitrogen atom.

However, in the case of the initial presence of defects in the structure, the embedding of oxygen into the lattice and the “healing” of the defect will be spontaneous in all ranges of temperature and source of oxygen (molecular or atomic oxygen and water).

Using an atomic oxygen source does not change the situation; in all temperature ranges, the most favorable process of oxidation of hexagonal boron nitride is the embedding of O in the lattice with removal of the nitrogen atom or healing of the existing defects (see Figure 1b).

In the presence of molecular hydrogen in the system, oxygen can be deposited as an −OH group. At ~50 K, this process becomes endothermic, and oxygen is more likely to precipitate without dissociation as a peroxide.

### 3.3. Electronic Properties of h−B_x_N_y_O_z_ Structures

**Figure 3:**
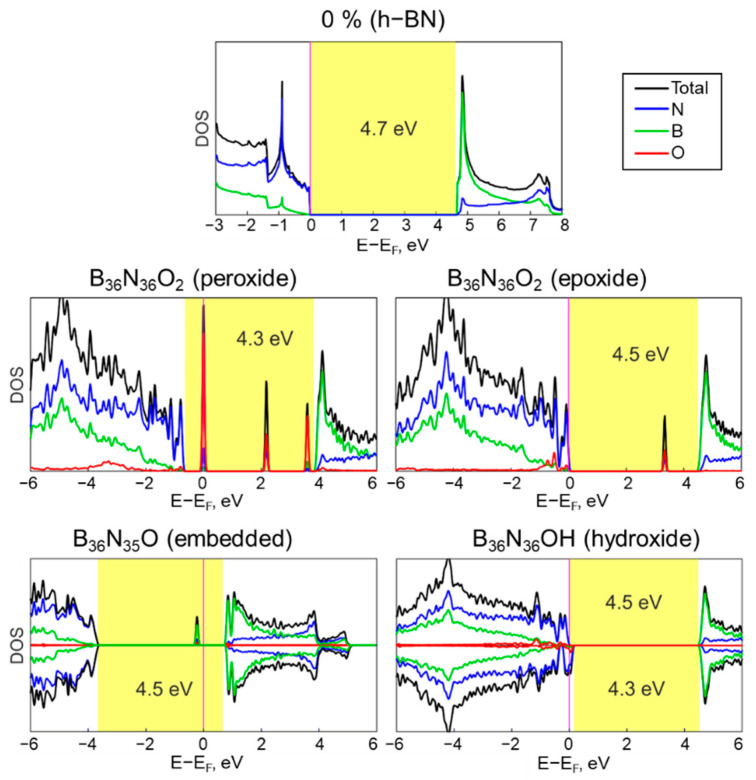
Density of electronic states of B_x_N_y_O_z_ structures. Boron, nitrogen, and oxygen contributions as marked by green, blue, and red colors, respectively. The band gap is shaded by yellow. The Fermi level is vanished and marked by vertical line.

The band-gap width and the presence of impurity levels in wide-gap insulators, such as hexagonal boron nitride, play a crucial role in its optical and electronic properties. Introducing defects or adsorption of reference atoms can significantly change the electronic properties of the material. Even dispersion interaction with other two-dimensional materials can significantly change the electronic properties of hexagonal boron nitride. Thus, when creating heterostructures of hexagonal boron nitride with graphene or phosphorene, h−BN will act as an electron donor [58], while interaction with InSe reverses its properties, making it an acceptor [59]. Moreover, oxygen adsorption can affect the electronic properties of h−BN because the formation of chemical bond between oxygen and h−BN sheet leads to introducing impurity levels in the h−BN band gap [38]. Moreover, it has been even suggested [24] that the electronic properties of the h−BN monolayer can be tuned by oxygen doping with a controllable change of the band gap down to 2.1 eV. In this regard, we studied the electronic properties of the stable structures by calculating the density of electronic states and band structures (Figure 3).

As shown in Figure 3, in the framework of the chosen approach, the pure h−BN monolayer displays band gap of 4.7 eV, which is consistent with previous theoretical studies [38]. The adsorption of oxygen-containing groups leads to the appearance of impurity levels and slight decreasing of the band gap value to 4.3–4.5 eV. The projected density of states (PDOS) by oxygen (red lines), boron (green lines), and nitrogen (blue lines) atoms show that the localized impurity states are mainly determined by the oxygen atoms and, to a lesser extent, by the neighboring boron or nitrogen atoms.

Since, in the peroxide and epoxy forms, the oxygen-containing groups form two bonds to the surface of the hexagonal boron nitride, no magnetic moment is observed in this case. In contrast, the embedding of oxygen or binding to a single OH group results in the appearance of slight magnetism in the B_x_N_y_O_z_ structure. The spin polarization is weakly detectable and is observed mostly at the bottom of the conduction band and in the presence of a spin-up impurity level. In contrast to surface adsorbed oxygen, the impurity level includes, to a greater extent, the contribution from boron atoms associated with the embedded oxygen. The magnetic moment for this structure is 1.0 *μ_B_* [60].

Adsorption of oxygen in the hydroxide form does not lead to the formation of impurity levels; however, it leads to the formation of slight semi-metallicity. The Fermi level appears to be below the top of the valence band for the spin-down states generated by the nitrogen and oxygen contributions.

These results confirm the fact that oxidation of boron nitride, even at low concentrations, can significantly affect the electronic and magnetic properties of the system, even leading to the appearance of semimetallic properties.

## 4. Conclusions

We investigated the oxidation of a two-dimensional h−BN sheet and the associated electronic properties of h−B_x_N_y_O_z_. Defectless h−BN strongly resists oxidation by pure oxygen or water molecules. The oxygen deposits only with a low concentration and only in a non−dissociated (peroxide) state to perfect lattice, which proves its high chemical stability. For example, at a temperature less than 1700 K at perfect h−BN, only adsorption of molecular oxygen without dissociation occurs, whereby this process occurs spontaneously only up to a temperature of 220 K. Only at higher temperature embedding of O into the lattice can be expected. This result additionally proves that h−BN can be utilized as a 2D anticorrosion coverage, as was shown in the number of experiments. The presence of structural defects allows easy oxygen embedding in all considered temperature ranges irrespective of oxygen source. Oxygenation allows to change the electronic and magnetic properties of the h−BN system.

The authors hope that this work allows a better understanding of the process of oxygen adsorption on h−BN and makes it possible to evaluate its temperature stability, which may help in its further use as an anticorrosion coating. Further development of the work may involve the study of more complex reactions involving nitrogen oxides and other reagents and products, as well as a particular oxidation mechanism. It would also be important to directly study the kinetics of process, which would allow better description of oxygenation.

## Data Availability

Not applicable.
